# Selective Increment of Synovial Soluble TYRO3 Correlates with Disease Severity and Joint Inflammation in Patients with Rheumatoid Arthritis

**DOI:** 10.1155/2020/9690832

**Published:** 2020-09-11

**Authors:** Julia Vullings, Juliana P. Vago, Claire E. J. Waterborg, Rogier M. Thurlings, Marije I. Koenders, Peter L. E. M. van Lent, Peter M. van der Kraan, Flavio A. Amaral, Fons A. J. van de Loo

**Affiliations:** ^1^Experimental Rheumatology, Department of Rheumatology, Radboud Institute for Molecular Life Sciences, Radboud University Medical Center, Nijmegen, Netherlands; ^2^Departamento de Bioquímica e Imunologia, Instituto de Ciências Biológicas, Universidade Federal de Minas Gerais, Belo Horizonte, Minas Gerais, Brazil

## Abstract

**Objective:**

To investigate the role of TAM receptors in rheumatoid arthritis (RA) by determining synovial tissue TAM receptor expression, synovial fluid levels of soluble TAM receptors, and the relationship between soluble TAM receptors, joint inflammation and disease activity.

**Methods:**

TAM receptor expression was determined by immunohistochemistry on the synovium from RA and osteoarthritis (OA) patients. Soluble (s) Tyro3, sAxl, sMer, and their ligand Gas6 were measured by ELISA in the synovial fluid of RA (*n* = 28) and OA (*n* = 12) patients and cytokine levels by multiplex immunoassay in RA samples. Correlation analyses were performed among sTAM receptors with local cytokine levels; systemic disease parameters like erythrocyte sedimentation rate (ESR), rheumatoid factor (RF), and anticyclic citrullinated peptide antibodies (ACPA); and disease activity scores (DAS28-ESR) in RA patients.

**Results:**

TAM receptors were expressed on different locations in the synovial tissue (lining, sublining, and blood vessels), and a similar expression pattern was observed in RA and OA patients. Synovial fluid sTyro3 and sMer were significantly enhanced in RA compared to OA patients, whereas no significant differences in sAxl and Gas6 levels were found. In RA samples, sTyro3 levels, but not sMer, correlated positively with proinflammatory local cytokines and the systemic factor erythrocyte sedimentation rate. Moreover, stratification analysis showed high sTyro3 levels positively correlated with higher DAS28-ESR and in RF and ACPA double positive RA patients.

**Conclusion:**

sTyro3 in the synovial fluid of RA patients correlates with local inflammatory molecules and systemic disease activity. These findings suggest that the reduced negative control of cell activation by TAM receptors due to their shedding in the synovial fluid, mainly sTyro3, favoring joint inflammation in RA patients.

## 1. Introduction

Rheumatoid arthritis (RA) is a common autoimmune disease marked by chronic and unrestrained inflammation, hyperplasia of synoviocytes, and damage of both the articular cartilage and bone. In the arthritic joint, the synovium is infiltrated by both innate and adaptive immune cells which, together with the proliferation of tissue-resident fibroblasts, leads to pannus tissue formation at the articular cartilage and bone interface. Overall, this eventually leads to damage and loss of articular cartilage matrix and bone tissue [1, 2]. Osteoarthritis (OA) is an age-related musculoskeletal disease characterized by progressive joint destruction, including breakdown of cartilage matrix [3]. At the synovial tissue level, the osteoarthritic joint shows a high degree of similarity with RA by increased presence of macrophages and lymphocytes. Although not an autoimmune disease, synovial inflammation, one of the hallmarks of RA, is often observed in OA [4–6]. The main difference, however, is the lack of neutrophils in OA joints, a cell that undergoes apoptosis and which clearance by TAM receptors mediates the resolution of several types of inflammation [7, 8].

The TAM receptor tyrosine kinase family consists out of Tyro3, Axl, and Mer (gene name MERTK). TAM receptors are expressed on, amongst others, monocytes, macrophages, and dendritic cells and play a critical role in natural anti-inflammatory feedback mechanisms and the phagocytosis of apoptotic cells [8–11]. The two principal TAM receptor protein ligands are Growth Arrest-Specific 6 (Gas6) and Protein S (Pros1). Gas6 is a ligand for all three TAM receptors but with the highest affinity for Axl, whereas Pros1 can only activate Tyro3 and Mer [12, 13]. TAM receptor ligands act as bridging molecules between one of the TAM receptors and phosphatidylserine (PS) that is expressed as an ‘eat-me signal' on the surface of apoptotic cells, thereby effectively opsonising apoptotic cells for TAM receptor-mediated efferocytosis [8, 12, 14]. In addition, activation of TAM receptors induces, among others, suppressor of cytokine signaling (SOCS) proteins 1 and 3, which reduce production of numerous cytokines [9, 14, 15]. Although we recently showed that Mer and Axl play a protective role in mouse models of RA, the exact function of TAM receptors in RA patients remains largely unknown [16–18]. TAM receptors can be cleaved from the cell surface, leading to shedding of their soluble ectodomain and consequently a soluble form of the receptor [19, 20]. These soluble TAM (sTAM) receptors may inhibit the immune regulatory and anti-inflammatory effects of TAM receptor activation by reducing membrane-bound receptors and by neutralization of TAM ligands by acting as decoy receptors [21, 22]. Soluble Axl (sAxl) mainly binds Gas6, whereas soluble Tyro3 (sTyro3) has the highest affinity for Pros1. However, Tsou et al. showed that soluble Mer (sMer) is not able to bind TAM receptor ligands and can therefore not act as a decoy receptor [13]. Elevated sMer and sAxl plasma levels are observed in patients with systemic lupus erythematosus (SLE), and these levels positively correlate with disease activity, inflammatory processes, and nephritis [23–25]. In addition, elevated plasma sMer levels correlate with disease activity in patients with Sjögren's syndrome [26].

To date, nothing has been described about locally produced sTAM receptor levels in RA patients, and the function of sTAM receptors in this disease remains largely unknown. Therefore, in the present study, we investigated the expression of TAM receptors in the synovial tissue and levels of sTAM receptors in the synovial fluid of RA patients using OA samples as the control. In addition, we analyzed the relationship between synovial fluid sTAM receptor levels and cytokine levels, systemic disease parameters, and disease activity scores in RA patients.

## 2. Materials and Methods

### 2.1. RA and OA Patients

RA (*n* = 28) (21 females, 7 males, mean age: 58.3 ± 14.9 years) and knee OA (*n* = 12) (7 females, 5 males, mean age: 56.8 ± 7.5 years) patients were recruited at the department of Rheumatology (Radboud University Medical Center, Nijmegen, the Netherlands) and the Sint Maartenskliniek (Nijmegen, the Netherlands). In addition to gender and age, the following laboratory parameters of RA patients were recorded: erythrocyte sedimentation rate (ESR) and C-reactive protein (CRP). In the RA patient group, 16 out of 28 patients (57%) were double positive for rheumatoid factor (RF) and anticyclic citrullinated peptide antibody (ACPA) (>10 IU/ml), 2 were single RF positive and 3 were single ACPA positive, whereas 7 out of 28 RA patients (64%) were RF and ACPA double negative (<10 IU/ml) (25%). For 11 RA patients, disease activity scores were determined. Disease activity scores were calculated using the 28-Joint Disease Activity Score-erythrocyte sedimentation rate (DAS28-ESR) with three variables based on assessment of 28 joints and ESR, according to the recommendations from the European League against Rheumatism (EULAR) [27]. Disease activity scores below 3.2 indicated low disease activity, whereas a score between 3.2 and 4.0 and a score above 4.0 represented medium and high disease activity, respectively. Clinical and demographic characteristics of RA and OA patients are presented in Table 1.

### 2.2. Human Synovial Fluid

The synovial fluid from RA and OA patients was obtained during consultations at the polyclinic to alleviate pressure and pain of knee joints caused by edema or swelling (synovial inflammation). All material was considered surplus material; therefore, ethical approval was not required. Procedures were performed in accordance to the Dutch code of conduct for responsible use of human tissue in medical research (https://www.federa.org/code-goed-gebruik). Written informed consent was obtained from all patients. Upon collection, the synovial fluid was centrifuged at 1,700 x g for 10 minutes at 4°C, followed by 30 minutes at 10,000 x g at 4°C to remove cells. The supernatant was aliquoted and stored at -80°C. To reduce viscosity, synovial fluid samples were thawed and treated with 75 U/ml of hyaluronidase (H3506; Sigma-Aldrich, Saint Louis, MO, USA) for 15 minutes at 37°C and subsequently centrifuged at 1,000 x g for 10 minutes at 4°C. Samples were aliquoted and stored at -20°C until further analysis.

### 2.3. Detection of Synovial Fluid sAxl, sMer, sTyro3, and Gas6 Levels by Enzyme-Linked Immunosorbent Assay (ELISA)

Synovial fluid sAxl (DY154), sMer (DY6488), sTyro3 (DY859), and Gas6 (DY885B) concentrations were determined using the DuoSet sandwich ELISA kits purchased from R&D Systems (Minneapolis, MN, USA). All ELISAs were performed according to the manufacturer's instructions using the DuoSet ELISA Ancillary Reagent Kit 2 (DY008; R&D Systems). In case of detection of sAxl and Gas6, synovial fluid samples were diluted 30 times, whereas for the detection of sMer and sTyro3, samples were diluted 10 and 5 times, respectively. Synovial fluid samples were diluted in Reagent Diluent (DY995; R&D Systems). Absorbance at 450 nm with a correction wavelength of 540 nm was detected using a microplate reader (CLARIOstar, BMG LABTECH).

### 2.4. Detection of Synovial Fluid Cytokine Levels by Multiplex ELISA

Cytokines in the synovial fluid of RA patients were measured on a Bio-Plex 200 system using a magnetic bead-based multiplex immunoassay (Bio-Rad Laboratories, Hercules, CA, USA). The synovial fluid was diluted 1 : 4 with Bio-Plex sample diluent (10014641; Bio-Rad Laboratories). The assay was performed according to protocols specified by the manufacturer and with the reagents (diluents, calibrators, blocking reagents, and detecting-antibody mixtures) included with their kits. Data analysis was performed with Bio-Plex Manager software (Bio-Rad Laboratories).

### 2.5. Immunohistochemistry of TAM Receptors on Human Synovial Tissue

RA and OA synovial tissues obtained from the knee joint during surgery at the Radboud University Medical Center (Nijmegen, the Netherlands) were used to determine protein expression of Axl, Mer, and Tyro3. This material was considered surgery surplus material. Procedures were performed in accordance to the Dutch code of conduct for responsible use of human tissue in medical research (https://www.federa.org/code-goed-gebruik). Paraffin-embedded synovial tissue sections were deparaffinized and rehydrated. Antigen retrieval was performed in Tris/ethylenediamine tetraacetic acid (EDTA) buffer (pH 9) heated to 60°C for Mer and citrate buffer (pH 6) heated to 60°C for Axl and Tyro3. Endogenous peroxidase was blocked by 3% hydrogen peroxide. Sections were blocked with 10% normal goat serum and 1% bovine serum albumin (BSA) in TBS for 20 minutes at RT before incubation with rabbit anti-human Axl (1 : 600; C89E7; Cell signaling, Danvers, MA, USA), rabbit anti-human Mer (1 : 2000; ab52968; Abcam, Cambridge, UK), rabbit anti-human Tyro3 (1 : 500; ab109231; Abcam), or rabbit anti-human IgG (1 : 74000; X0936; Agilent Technologies, Santa Clara, CA, USA) overnight at 4°C. Subsequently, sections were incubated with biotinylated goat anti-rabbit IgG (1 : 400; PK-6101; Vector Laboratories, Peterborough, UK) for 30 minutes at RT. A biotin-streptavidin horseradish peroxidase detection system was used according to manufacturer's protocol (PK6101; Vector Laboratories). Bound complexes were visualized with diaminobenzidine (DAB) by incubation for 10 minutes at RT. All antibodies were diluted in 1% BSA in TBS. Sections were counterstained with hematoxylin. Pictures were taken with the Leica DMR microscope (Leica Microsystems, Wetzlar, Germany) at 20x and 40x magnification.

### 2.6. Statistical Analysis

All data were analyzed with GraphPad Prism Software (version 5.03, San Diego, CA, USA). Data were shown as dot plots with mean or as correlations. Comparisons between groups were performed using Student's unpaired *t*-tests or by covariance analysis (Tukey's multiple comparison test for post hoc test). Correlations between continuous data were assessed using Pearson's correlation coefficient. *P* values lower than 0.05 were considered statistically significant.

## 3. Results

### 3.1. Differential Synovial Expression of TAM Receptors in RA

**Figure 1:**
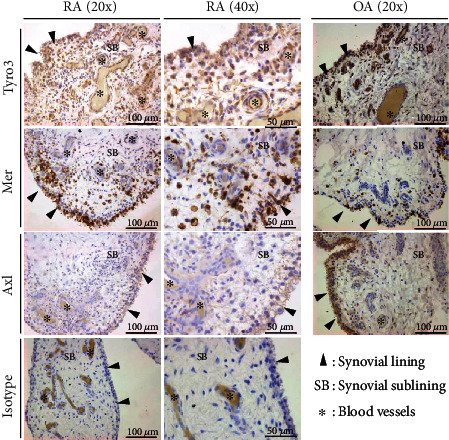
Immunohistochemistry of TAM receptors in the human synovium from rheumatoid arthritis and osteoarthritis patients. Synovial biopsies from rheumatoid arthritis (RA) (*n* = 2) and osteoarthritis (OA) (*n* = 8) patients were processed for immunohistochemical staining of Tyro3, Mer, Axl, and IgG isotype control. Sections were counterstained with hematoxylin. Pictures were taken at 20x and 40x magnification.

Immunohistochemistry on the synovial tissue from RA patients revealed differences in expression of TAM receptors among different compartments in the synovium (Figure 1). Tyro3-positive cells were identified mainly in lining cells, instead sublining cells. In addition, expression of Tyro3 was also observed in association with blood vessels, in particular endothelial cells. On the other hand, vascular endothelial cells were Mer negative, whereas Mer-positive cells were observed in both the lining and sublining. Regarding the Axl expression, only cells from the synovial lining were positive, without staining in the sublining or blood vessels. The synovial tissue from OA patients was used as control, while with similar compartmentalized expression of all three TAM receptors.

**Figure 2:**
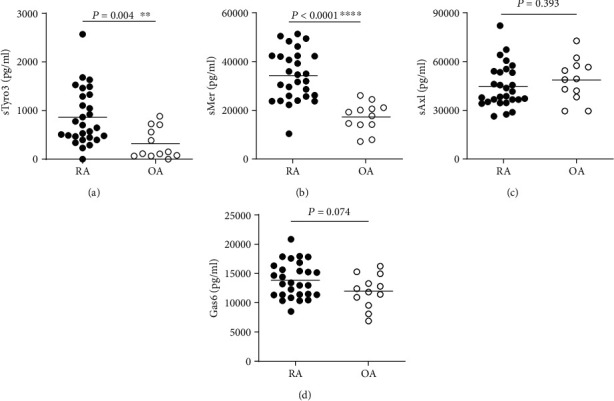
Soluble TAM receptors and Gas6 levels in the synovial fluid of rheumatoid arthritis and osteoarthritis patients. Soluble Tyro3 (sTyro3) (a), soluble Mer (sMer) (b), soluble Axl (sAxl) (c), and Gas6 (d) levels in the synovial fluid of rheumatoid arthritis (RA) (*n* = 28) and osteoarthritis (OA) (*n* = 12) patients were detected by ELISA. Data are presented as dot plots with mean tested by unpaired *t*-tests. ^∗∗^*P* < 0.01; ^∗∗∗∗^*P* < 0.0001.

**Figure 3:**
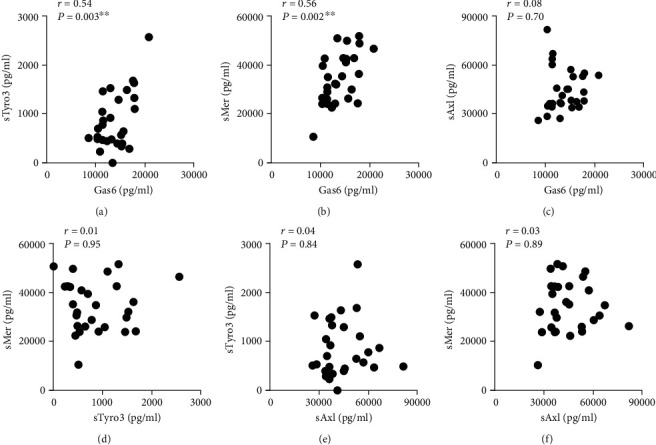
Relationship between soluble TAM receptor levels in the synovial fluid of rheumatoid arthritis patients. Relationship between soluble Tyro3 (sTyro3), soluble Mer (sMer), soluble Axl (sAxl), and Gas6 levels in synovial fluid of rheumatoid arthritis patients (*n* = 28). Correlations are depicted for sTyro3–Gas6 (a), sMer–Gas6 (b), sAxl–Gas6 (c), sMer–sTyro3 (d), sTyro3–sAxl (e), and sMer–sAxl (f). Data are presented as the Pearson *r* value (*r*) and *P* value (*P*) for each correlation. ^∗∗^*P* < 0.01.

To study if sTAM receptor levels were increased in synovial fluid of RA patients, sTyro3, sMer, sAxl, and Gas6 levels were determined by ELISA. In RA patients, both sTyro3 and sMer levels were elevated compared to OA patients (*P* = 0.0042 and *P* < 0.0001, respectively) (Figures 2(a) and 2(b)). However, no significant differences in sAxl and Gas6 levels were observed between RA and OA patients (*P* = 0.3932 and *P* = 0.0740, respectively) (Figures 2(c) and 2(d)). In fact, sAxl and Gas6 levels were comparable to levels found in plasma of both RA patients and healthy controls (data not shown). There were positive correlations between sTyro3 and sMer with the ligand Gas6 in the synovial fluid (Figures 3(a) and 3(b)). On the other hand, no significant correlations were found between sAxl with Gas6 nor among sMer, sAxl, and sTyro3 in the synovial fluid of RA patients (Figures 3(c) – 3(f)). Of note, there were no differences in sTyro3 and sMer levels in the synovial fluid between males and females (Supplementary Fig. S1) neither the observed increases in sTyro3 nor sMer in the synovial fluid were influenced by the age of the patients (Supplementary Fig. S2).

### 3.2. sTyro3 Positively Correlated with Proinflammatory Cytokine Levels in RA Synovial Fluid

**Figure 4:**
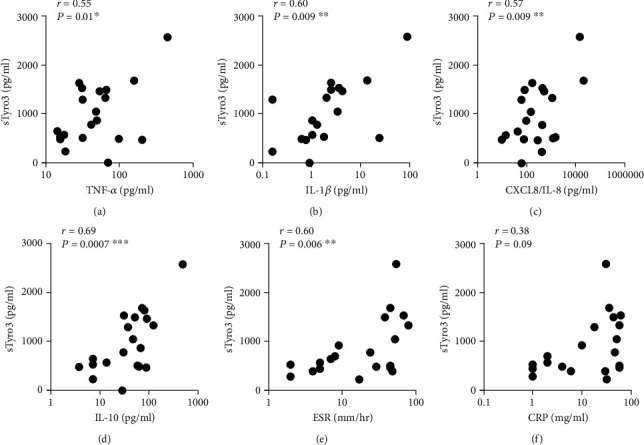
Relationship between soluble Tyro3 levels and inflammatory markers in the blood and synovial fluid of RA patients. Relationship between soluble Tyro3 (sTyro3) and the cytokines TNF-*α*, IL-1*β*, IL-8, and IL-10 in the synovial fluid of rheumatoid arthritis patients (*n* = 20). Correlations are depicted for sTyro3–TNF-*α* (a), sTyro3–IL-1*β* (b), sTyro3–IL-8 (c), and sTyro3–IL-10 (d). Relationship between soluble Tyro3 (sTyro3) and erythrocyte sedimentation rate (ESR) and C-reactive protein (CRP) in the blood of rheumatoid arthritis patients (*n* = 20). Correlations are depicted for sTyro3–ESR (e) and sTyro3–CRP (f). Data are presented as Pearson *r* value (*r*) and *P* value (*P*) for each correlation. ^∗^*P* < 0.05; ^∗∗^*P* < 0.01; and ^∗∗∗^*P* < 0.001.

As membrane-bound TAM receptors are involved in natural anti-inflammatory feedback mechanisms [8], the presence of high concentration of sTAM in the synovial fluid, particularly sTyro3 and sMer (as shown in Figure 2), could be associated with increased inflammation in RA joints. Thus, we next investigated if increased amounts of sTAM receptors coincide with the presence of key inflammatory molecules in RA joints. Indeed, sTyro3 levels positively correlated with synovial fluid levels of TNF-*α* (*r* = 0.55, *P* = 0.01), IL-1*β* (*r* = 0.60, *P* = 0.009), CXCL8/IL-8 (*r* = 0.57, *P* = 0.009), and IL-10 (*r* = 0.69, *P* = 0.007) (Figures 4(a) – 4(d)), but not with IL-6 (*r* = 0.22, *P* = 0.36) (Table 2) in RA patients. In addition to local inflammatory markers, sTyro3 levels also positively correlated with erythrocyte sedimentation rate (ESR) (*r* = 0.60, *P* = 0.006) (Figure 4(e)) and weakly however not significantly with CRP levels (*r* = 0.38, *P* = 0.09) (Figure 4(f)), both systemic measures of inflammation. In contrast, no significant correlations were found between sMer and local cytokine levels or ESR and CRP blood levels (Table 2). Similar to sTyro3, Gas6 in the synovial fluid also positively correlated with most of those molecules, except by IL-6, ESR, and CRP (Table 2). Thus, the presence of sTyro3 in the synovial fluid suggests a reduced control of joint inflammation in RA, impacting on the worsening of the disease.

### 3.3. sTyro3 Levels in RA Synovial Fluid Correlated with Systemic Disease Parameters and Disease Activity Scores

**Figure 5:**
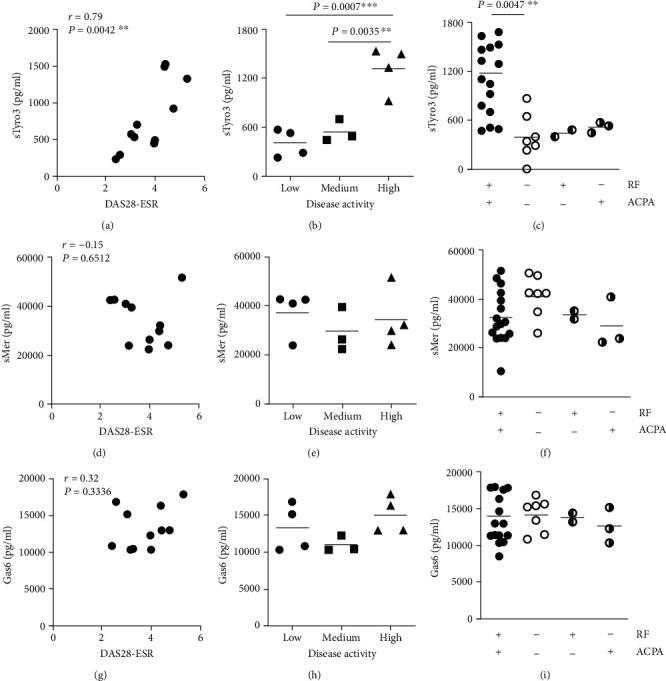
Relationship between soluble Tyro3 levels, disease activity scores, and systemic disease parameters. Relationship between soluble Tyro3 (sTyro3), soluble Mer (sMer), and Gas6 levels in synovial fluid of RA patients (*n* = 11) and disease activity scores based on erythrocyte sedimentation rate (DAS28-ESR) (a, d, g). sTyro3 levels in patients with low (<3.2), medium (3.2–4.0), and high (>4.0) DAS28ESR scores (b, e, h). Differences between soluble Tyro3 (sTyro3) in the synovial fluid of RA patients double positive (>10 IU/ml) for rheumatoid factor (RF) and anticyclic citrullinated peptide antibody (ACPA) (16 samples), single positive for RF (2 samples) or ACPA (3 samples), and double negative (<10 IU/ml) (7 samples) (c, f, i). Data are presented as *P* value for each comparison (unpaired *t*-tests) or as Pearson *r* value (*r*) and *P* value (*P*) for correlation. ^∗∗^*P* < 0.01; ^∗∗∗^*P* < 0.001.

Apart from an association with inflammation, it was also investigated if sTyro3 levels in the joints correlated with systemic disease parameters and disease activity scores. Indeed, sTyro3 levels demonstrated a significant correlation to DAS28-ESR scores (*r* = 0.79, *P* = 0.004) (Figure 5(a)) and sTyro3 levels in patients with a high disease activity (>4.0) were significantly higher than those with a low (<3.2) or medium (3.2-4.0) DAS28-ESR score (*P* = 0.0006) (Figure 5(b)). Furthermore, sTyro3 levels were significantly higher in rheumatoid factor (RF) and anticitrullinated protein antibody (ACPA) double positive patients compared to double negative patients (*P* = 0.005) (Figure 5(c)). Due to the low number of single RF (3) and ACPA (2) positive RA patients enrolled in this study, the statistical analysis was impaired, although their mean sTyro3 values were comparable to double negative RA patients. No significant correlations were found between sMer and DAS28-ESR (Figure 5(d)), disease activity (Figure 5(e)) or RF, and ACPA (Figure 5(f)), as well as Gas6 (Figures 5(g)–5(i)). Together, these results suggested that sTyro3 correlates with inflammation associated mechanisms and disease activity of RA.

## 4. Discussion

In this study, we showed that, in RA patients, local sTyro3 and sMer levels were increased as compared to OA patients, whereas sAxl and Gas6 levels in both RA and OA patients were within the normal plasma level range as found in healthy controls. In addition, elevated synovial fluid levels of sTyro3 were associated with local inflammatory mediators, systemic disease parameters, and disease activity scores, indicating a role for sTyro3 in RA pathology.

Proper efferocytosis during homeostasis or inflammation has been shown to restrict subsequent immune responses. This effect is pivotal in the maintenance of the immune tolerance. The role of TAM receptors has been described in various autoimmune diseases, including RA, multiple sclerosis (MS), and systemic lupus erythematosus (SLE) [28, 29]. In the context of RA, the expression of Axl has already been described in joints of patients [30]. Also, a recent study using a murine model of K/BxN serum-transfer arthritis showed that Axl was expressed by a distinct subset of CX_3_CR1^+^ tissue-resident macrophages, which form an internal immunological barrier at the synovial lining [31]. These findings reinforce our data regarding the detection of Axl in lining cells. In addition, to the best of our knowledge, this is the first work that describes the expression of Tyro3 and Mer in the human synovial tissue of RA patients.

Although measured in plasma, Xu et al. found a comparable result of elevated sTyro3 and sMer in RA compared to OA patients and that only sTyro3 levels linked to some clinical RA features, although correlations were weak [32]. In addition, Wu et al. reported elevated sTyro3 and sMer plasma levels in RA patients compared to healthy controls, while plasma sTyro3 did not correlate with clinical features of RA [23]. In our study, using the synovial fluid rather than plasma, we found strong positive correlations between sTyro3 levels in both inflammation and disease activity scores, indicating that the synovial fluid sTyro3 is a more reliable measure of joint inflammation in RA compared to plasma sTyro3 levels. Although the focus of our study was the analysis of local TAM expression, not in plasma, we believe our data are complementary and reinforce the findings from the published studies so far [23, 32]. Interestingly, changes in sTAM levels are not consistent when comparing different arthritis related autoimmune diseases. Elevated plasma levels of both sMer and sAxl were reported in SLE patients [23–25], whereas Sjörgen's syndrome patients presented elevation of only sMer [26]. In SLE and Sjögren's syndrome, sMer rather than sTyro3 levels seem to be associated with inflammation and disease activity [23, 24, 26], indicating that sTyro3 could be more specific for RA disease pathology.

TAM receptors could be differentially regulated, expressed on different cell types, as demonstrated in this study, or exhibit different functions in distinct autoimmune diseases [14]. This could explain the difference in the levels of soluble TAM receptors observed here as well as the lack of correlation among them. Thus, additional studies regarding the modulation of TAM receptors in the context of arthritis are needed. In addition, it has been described that inflammatory autoimmune diseases are often characterized by deregulated metalloproteinase activities [33], indicating that changes in the levels of sTAM receptors observed in RA patients could indeed be caused by differential activation of shedding mechanisms. For example, shedding of TAM receptors can decrease TAM-mediated anti-inflammatory signaling and thereby increase cytokine production by both reducing the amount of membrane-bound TAM receptors and by acting as a decoy receptor, capturing the TAM receptors ligands and thereby reducing activation of membrane-bound receptors [21, 22, 34]. The metalloproteinase ADAM17 has been described as a key enzyme responsible for the proteolytic cleavage of TAM receptors [19, 20, 34]. Although most studies were performed in mice, there are some indications that ADAM17 is also responsible for cleavage of TAM receptors in humans. For instance, patients with chronic kidney disease have increased plasma levels of sMer and sAxl with increased expression of ADAM17 on monocytes [35]. In addition, different works have already shown that ADAM17 is expressed higher in the serum and synovial fluid or tissue of RA compared to OA patients [36–38]. Thus, higher levels of synovial sTAM in RA, particularly sTyro3, impair an important endogenous anti-inflammatory branch to control joint inflammation.

The decreased membrane bound Tyro3 on synovial cells could have a direct effect in reducing the control of joint inflammation, favoring the positive correlation between sTyro3 and local inflammatory parameters or disease activity in RA. However, much of the knowledge about the anti-inflammatory properties of Tyro3 came through triple TAM receptor knock out studies, although few studies explore individual anti-inflammatory properties of Tyro3. For instance, neutralizing anti-Tyro3 antibody reverted the effect of Pros1 in the reduction of proinflammatory cytokines by the human gingival epithelial cell line stimulated with *Porphyromonas gingivalis* LPS [39]. Thus, the reduced availability of joint TAM receptor ligands through the scavenger property of sTyro3 might have a stronger impact in a defective control of joint inflammation in RA. At this point, TAM receptor ligands, particularly Gas6, are involved in the resolution of inflammation [40] and present protective effects in experimental arthritis [41]. On the other hand, sMer barely binds to TAM receptor ligands [13], weakening its contribution as a decoy receptor. For that reason, the effects of reduced Mer surface expression due to its shedding to the synovial fluid were not enough to reduce anti-inflammatory feedback, explaining why no correlation was observed between sMer and cytokine levels or disease activity.

sTyro3 and sAxl act as effective ligand antagonists by blocking Pros1 and Gas6, respectively, while sMer shows weak inhibitory activities toward both ligands [13]. Here, we showed that sTyro3 levels were associated with disease phenotype, as RF and ACPA double positive patients exhibited higher sTyro3 levels. Previous studies have demonstrated that Gas6 and Pros1 limit the immune response by inhibition of DC maturation and reduction of antigen presentation to T cells [42, 43]. The pronounced disease activity associated with RF and ACPA double positive patients could be explained by the blocking of TAM ligands due to elevated sTyro3 in RA patients. Although we have not seen a significant increase of Gas6 in the synovial fluid of RA patients, there was a trend for its increase (*P* = 0.074, Figure 2(d)). In experimental arthritis, Ruiz-Heiland et al. found increased levels of circulating Gas6 after induction of a murine model of arthritis [44], which can be speculated as an attempt to control tissue inflammation. Thus, this could explain the positive correlations between Gas6 and sTyro3 and sMer. On the other hand, increased local soluble TAM receptors could scavenge TAM ligands, exemplified here only by Gas6. Thus, reduced availability of Gas6 would impair the anti-inflammatory function of this molecule due reduced membrane TAM receptor activation. In fact, the protective role of both TAM receptor ligands Gas6 and Pros1 has already been described by our group in murine models of arthritis [16, 17, 41]. In these studies, Gas6 and Pros1 overexpression decreased arthritis severity, by reducing inflammation and by inhibiting the expression of proinflammatory cytokines. Although we have not measured Pros1 in this study, we believe that high levels of sTyro3 in the synovial fluid could indeed scavenge both TAM ligands, reducing their anti-inflammatory function in tissue inflammation in RA.

In summary, in the present study, we showed that sTyro3 and sMer levels are increased in the synovial fluid of RA patients, whereas only sTyro3 correlates with inflammation and disease parameters. This indicates a role for sTyro3 in RA disease pathology, especially in RF- and ACPA-positive patients. Further functional studies are needed to elucidate the role of sTyro3 in the development of RA and to investigate whether sTyro3 is a marker that will be helpful in the evaluation of RA disease activity and local inflammation.

## Figures and Tables

**Table 1:** Demographic and clinical characteristics of rheumatoid arthritis (RA) and osteoarthritis (OA) patients.

Characteristics	RA patients (*n* = 28)	OA patients (*n* = 12)
Age, years (mean, S.D.)	58.3 ± 14.9	56.8 ± 7.5
Female (*n*, %)	21, 75	7, 58
Male (*n*, %)	7, 25	5, 42
RF^+^ (*n*, %)^a^	19, 68	—
ACPA^+^ (*n*, %)^b^	18, 64	—
ESR in mm/h (mean, S.D.)	29.3 ± 24.2	—
CRP in mg/ml (mean, S.D.)	26.7 ± 23.8	—
DAS28-ESR (mean, S.D.)	3.75 ± 0.94	—

ACPA: anticyclic citrullinated peptide antibodies; CRP: C-reactive protein; DAS: disease activity score; ESR: erythrocyte sedimentation rate; OA: osteoarthritis; RA: rheumatoid arthritis; RF: rheumatoid factor. ^a^RF ^+^: >10 IU/ml. ^b^ACPA^+^: >10 IU/ml.

**Table 2:** Relationship between soluble TAM receptor levels and local and systemic inflammatory markers.

RA	TNF-*α*		IL-1*β*		IL-6		IL-8		IL-10		ESR		CRP	
	*r*	*P* value	*r*	*P* value	*r*	*P* value	*r*	*P* value	*r*	*P* value	*r*	*P* value	*r*	*P* value
sTyro3	0.555	0.011^∗^	0.597	0.009^∗∗^	0.216	0.361	0.567	0.009^∗∗^	0.695	0.001^∗∗∗^	0.595	0.006^∗∗^	0.396	0.084
sMer	0.231	0.328	0.115	0.649	-0.388	0.091	-0.011	0.963	0.230	0.199	0.057	0.810	-0.097	0.684
sAxl	0.325	0.162	-0.025	0.922	-0.397	0.083	0.129	0.589	0.168	0.479	-0.012	0.961	-0.141	0.552
Gas6	0.477	0.033^∗^	0.481	0.043^∗^	-0.083	0.727	0.529	0.017^∗^	0.562	0.01^∗∗^	0.223	0.345	-0.034	0.887

Relationship between soluble Mer (sMer), soluble Tyro3 (sTyro3), soluble Axl (sAxl), and Gas6 and cytokine levels in the synovial fluid and erythrocyte sedimentation rate (ESR) and C-reactive protein (CRP) in the blood of rheumatoid arthritis (*n* = 20). Data are presented as Pearson *r* value (*r*) and *P* value for each correlation. ^∗^*P* < 0.05; ^∗∗^*P* < 0.01; and ^∗∗∗^*P* < 0.001.

## Data Availability

All relevant data are within the paper and its supporting information files.

## Additional Points

*Key message*. TAM receptors are expressed on different locations in synovial tissue (lining, sublining, blood vessels). Soluble Tyro3 and soluble Mer levels are increased in synovial fluid of RA patients compared to OA patients. In RA patients, soluble Tyro3 in synovial fluid positively correlates with joint inflammation and disease activity.

## Supplementary Materials

Supplementary Materials Figure S1: soluble TAM receptor levels in synovial fluid of male versus female rheumatoid arthritis. Soluble Tyro3 (sTyro3) (A), soluble Mer (sMer) (B), soluble Axl (sAxl) (C), and Gas6 (D) levels in the synovial fluid of male versus female rheumatoid arthritis patients (*n* = 28). Data are presented dot plots with mean unpaired *t*-tests. Figure S2: relationship between soluble TAM receptor levels and age. Relationship between soluble Tyro3 (sTyro3) (A), soluble Mer (sMer) (B), soluble Axl (sAxl) (C), and Gas6 (D) levels in the synovial fluid and the age in years of rheumatoid arthritis patients (*n* = 28). Data are presented as the Pearson *r* value (*r*) and *P* value (*P*) for each correlation.
