# Supplementary figures and images for: Selective Increment of Synovial Soluble TYRO3 Correlates with Disease Severity and Joint Inflammation in Patients with Rheumatoid Arthritis
Source: J Immunol Res. 2020 Sep 11;2020:9690832. doi: 10.1155/2020/9690832 (PMC7502136; doi:10.1155/2020/9690832)

**Supplementary Figure 1**


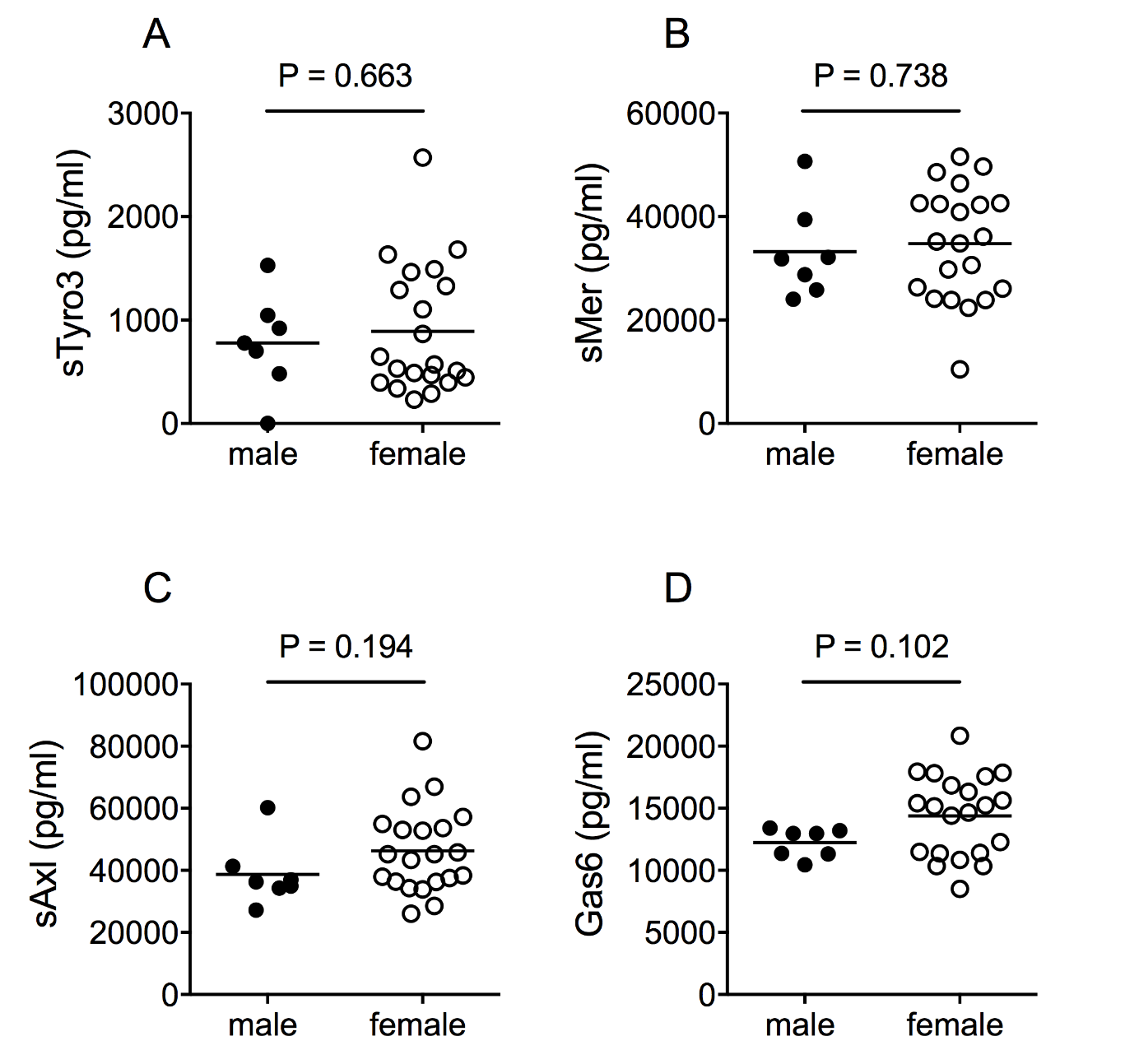


**Supplementary Figure 2**


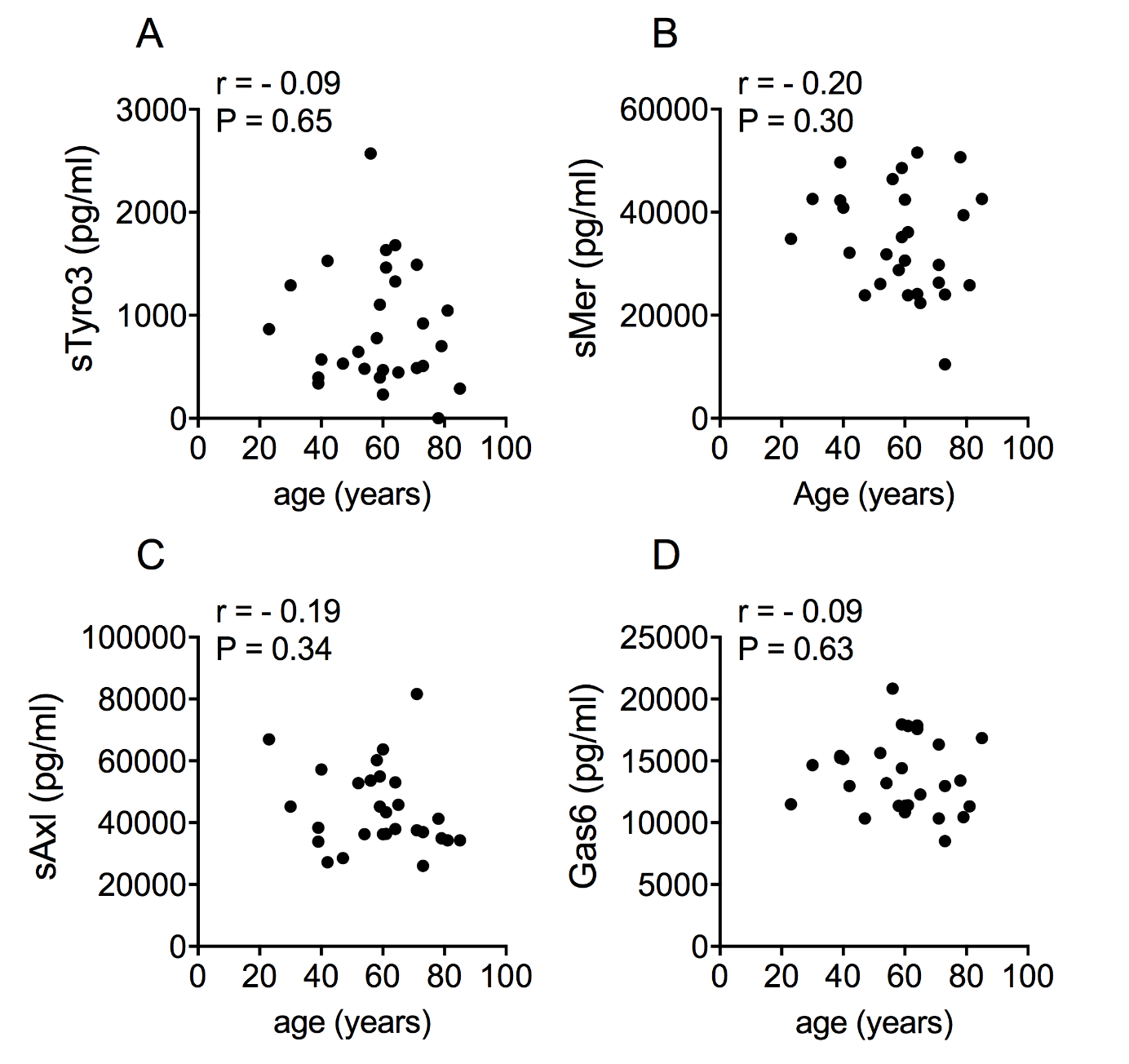

Supplement: Supplementary Materials — Figure S1: soluble TAM receptor levels in synovial fluid of male versus female rheumatoid arthritis. Soluble Tyro3 (sTyro3) (A), soluble Mer (sMer) (B), soluble Axl (sAxl) (C), and Gas6 (D) levels in the synovial fluid of male versus female rheumatoid arthritis patients (n = 28). Data are presented dot plots with mean unpaired t-tests. Figure S2: relationship between soluble TAM receptor levels and age. Relationship between soluble Tyro3 (sTyro3) (A), soluble Mer (sMer) (B), soluble Axl (sAxl) (C), and Gas6 (D) levels in the synovial fluid and the age in years of rheumatoid arthritis patients (n = 28). Data are presented as the Pearson r value (r) and P value (P) for each correlation. [file 9690832.f1.docx]
